# A review of the effect of the light environment of the VDT workspace on the “learning to learn” effect of video game training

**DOI:** 10.3389/fnins.2023.1093602

**Published:** 2023-02-24

**Authors:** Xiang Cheng, Yonghong Yan, Tao Hu, Yinghui Lv, Yue Zeng

**Affiliations:** ^1^Faculty of Architecture and Urban Planning, Chongqing University, Chongqing, China; ^2^Key Laboratory of the Ministry of Education of Mountainous City and Towns Construction and New Technology, Chongqing University, Chongqing, China

**Keywords:** VDT workspace, light environment, VDT display lighting, video game training, learning transfer, learning to learn

## Abstract

In recent years, the role of video games in enhancing brain plasticity and learning ability has been verified, and this learning transfer is known as the “learning to learn” effect of video game training. At the same time, against the background of healthy lighting, the influence of non-visual effects of light environment on the human rhythmic system has been gradually confirmed. As a special operation form of Visual Display Terminal (VDT) operation, video game training has a high dependence on VDT equipment and the VDT screen, and the background usually has a huge difference in brightness. Compared with the light environment of ordinary operation space, the light environment of VDT operation space is more complex. This complex light environment's non-visual effects cause human emotions, alertness, fatigue, cognitive ability, and other changes, which may affect the efficiency of the “learning to learn” effect of video game training. This article focuses on the impact of the light environment in the VDT workspace on the “learning to learn” effect of video game training. It first traces the factors that trigger the “learning to learn” effect of video game training, that is, the improvement of people's attention, perception, and cognitive ability. Then, the influencing mechanism and the evaluation method of the VDT workspace space light environment on the human rhythm system are discussed based on the basic theory of photobiological effect. In addition, the VDT display lighting light time pattern, photophysical properties, regulation, and protection mechanism on the human rhythm system are studied to demonstrate the VDT workspace light environment's special characteristics. Finally, combined with the progress of artificial lighting technology and the research results of health lighting, given the “learning to learn” effect of video game training, some thoughts on the design of the light environment of the workplace and future research directions are presented.

## 1. Introduction

Over the past 30 years, video games have evolved from a reasonably niche hobby to a pervasive part of modern culture (Bavelier and Green, 2019), especially in the entertainment and leisure sector. In terms of economic revenue, the video game industry has out-earned the film and music industries combined in each of the past 8 years (MYBOOSTING.GG, 2022). However, the benign development of the video game industry still faces severe challenges and questions. With the vigorous development of video games, the “game disorder” caused by excessive addiction of some groups to video games has caused many negative effects on physical and mental health. Aziz et al. (2021) found that gamers are more likely to suffer from obesity, and pain in various body parts and eye problems are also more frequent. In addition, there is a complex relationship between playing video games and mental health. For example, a large-scale study on children aged 7–11 (*N* = 2,442) reported that a large number of games (more than 9 h per week) were related to an increase in children's behavioral problems and a decrease in prosocial behaviors (Pujol and Fenoll, 2016). This conclusion has been confirmed by studies that it is also applicable to older children (10–15 years old) (Przybylski, 2014). Therefore, although games have met the entertainment and leisure needs of modern life and produced huge economic benefits, people's attitude toward them is still controversial.

In recent years, with the continuous exploration of the research on video games, their positive effects on people's productivity and life have begun to be explored more and more. A large number of studies have shown that video game training can not only improve the performance of the game itself but also improve the learning ability in non-game scenes (Chamberland and Michon, 2016; Bediou et al., 2018; Bavelier and Green, 2019; Zhang et al., 2021); this improvement may be related to the improvement of players' attention, perception, and cognition. While video games may have different effects on cognitive ability and neural perception due to factors such as individual differences or game types (Schenk et al., 2020), Kuhn et al. (2014) confirmed that even playing simple video games such as Super Mario can induce plasticity changes in the brain structure. In addition, although video games can help people train their brain function, recent studies (Chopin et al., 2019; Pasqualotto et al., 2022) suggest that this training effect often requires a certain training time (more than 20 h) to accumulate, and the benefits of some training can be maintained for a long time or even lasting (Bejjanki et al., 2014). Moreover, this improvement is not independent of each other. Some studies believe that better cognitive skills can promote the improvement of cognitive adaptability.

Video games that can endow people with the characteristic of “learning to learn” have begun to be used in various fields of daily life such as education and medical care. Interestingly, in the field of education, not only are video games designed for the explicit purpose of education but also many commercial games with enhanced cognitive ability have begun to be applied in the field of education. For example, Schenk et al. (2020) found that action game training can enhance attention control and this enhancement can further improve the classification learning ability in basic cognitive ability. In terms of perceptual tasks closely related to the improvement of learning efficiency, Zhang et al. (2021) found that habitual action video game players have higher learning efficiency than non-action video game players, and the learning transfer effect of action video game training can greatly improve the speed of learning other knowledge in the future. In addition, the application of video games in the medical field is also developing. Gambacorta et al. found that the action video game method can be used as an effective auxiliary treatment method for children's amblyopia, enabling children to achieve similar effects with traditional standard treatment in a shorter time. Sil et al. (2014) research explored the role of video games in alleviating patient suffering as well as studies beginning to design video games as a medium for young cancer patients to learn about their diseases and treatments (Kato et al., 2008). The above research not only explains the reasons for the rise of video games in the field of education and medical care in recent years but also foresees the broad development prospects of video games in these fields.

However, the architectural design standards related to the video game training environment are still relatively lacking. In fact, the quality of the indoor environment, such as indoor air quality, acoustic environment, light environment, and thermal environment are likely to affect the indoor comfort (Huang et al., 2012; Lin et al., 2018), and thus affect the effectiveness of the video game training. The proposal and rise of health lighting have confirmed that the light environment not only affects the indoor visual effect but also affects the human body's rhythm system through non-visual effects. The current light environment in video game training has, therefore, gradually attracted attention (Berson et al., 2002; Hattar et al., 2002). There are two main channels for light to affect health through the human eye. One is the visual channel of “Light—retina—optic chiasma—lateral geniculate body—cerebral cortex,” and the other is the non-visual channel of “Light—retina—hypothalamus—suprachiasmatic nucleus—pineal gland—melatonin—circadian rhythm” (Berson et al., 2002). The human circadian rhythm dominated by non-visual channels is mainly excited by the suprachiasmatic nucleus (SCN) located in the hypothalamus of the brain to form rhythmic oscillations (Kalsbeek et al., 2006). The visual and non-visual channels of light are not completely independent, and their interaction ultimately determines the combined effect of light on vision, emotion, and physiology, which in turn affects human rhythm, alertness, sleep quality, cognitive ability, etc. (Zhu and Duan, 2022). For example, studies have shown that environment with high light levels during the day or at night can improve personnel's alertness, reduce sleepiness, reduce fatigue, and have a faster reaction speed to tasks requiring attention (Badia et al., 1991; Grunberger et al., 1993; Dijk, 2008); Moreover, environments containing a certain dose of blue light can increase people's vitality, significantly reduce the characteristics of emotional depression (Zeng and Hao, 2016), and thus improve work efficiency (Partonen and Lonnqvist, 2000; Mills et al., 2007; Iskra-Golec et al., 2012). Therefore, the non-visual effects of the light environment are likely to affect the training performance of video games.

In addition, video game training or competition is a highly dependent form of operation on the Visual Display Terminal (VDT) equipment. In the workspace, there is not only environmental lighting (hereinafter referred to as environmental lighting) but also VDT screen display lighting (hereinafter referred to as VDT display lighting). Compared with the general VDT operation light environment, the more complex and changeable light environment of the VDT workspace may have multiple compound effects on the work efficiency and physical and mental health of operators (Ranasinghe et al., 2016). However, previous studies on the characteristics of “learning” in video game training mainly focused on the game type (Bediou et al., 2018), training time (Zhang et al., 2021), and the age of the player (Cardoso-Leite et al., 2021), etc. Although many effects of video game training have been confirmed, there are few studies on whether the game environment, that is, the light environment of the e-sports VDT operation space, affects the “learning to learn” effect of video game training.

It is worth noting that changes in mood, alertness, and fatigue induced by the non-visual effects of the light environment may be related to the effectiveness of the “learning to learn” effect of video game training on attention and perception, and both have effects on human cognitive abilities. How to accurately determine the key factors that affect the role of “learning” in video game training in such a complex, multi-dimensional, and interactive relationship, and how to control them, is a difficult problem in the research of the VDT workspace light environment. Thus far, there have been few relevant studies in domestic and foreign literature. However, against the backdrop of the growing popularity of video games and the upsurge of research on the gain effect of “learning to learn”, this problem has been unavoidable, and it is urgent to start relevant research. This article focuses on the impact of the VDT workspace light environment on the “learning to learn” effect of video game training. First, it examines the factors that trigger the “learning” effect of video game training, namely, the improvement of people's attention, perception, and cognitive ability. Then, through the analysis of the particularity of the light environment of the video game training space, the influencing mechanism and evaluation method of the VDT operation space light environment on the human body are discussed based on the basic theory of photobiological effect. Then, the influence of the lighting time mode, optical physical characteristics, adjustment, and protection mechanism of the VDT display lighting on this kind of light environment on the human body rhythm system is analyzed, and the influence of the light environment in VDT workspace on the “learning to learn” effect is demonstrated. Finally, combined with the progress of artificial lighting technology and research results on healthy lighting, this article discusses how to build a light environment that adds to the “learning to learn” effect of video game training.

## 2. Study of the “learning to learn” effect of video game training

Typically, learning a single task does not improve performance on other tasks, but in recent years, the effectiveness of using immersive media (e.g., video games) to enhance brain plasticity and learning capacity, and thus improve the performance of other tasks, has been demonstrated. These studies illustrate that video games can be used as a potential medium to train brain function, causing changes in attention, perception, and cognition, and enhancing performance on non-video game tasks through learning transfer effects, that is, the “learning to learn” effect.

### 2.1. Attention

Attention is the direction and concentration of human mental activities on certain things in the outside world and is associated with the learning efficiency of humans (Bavelier and Green, 2019). Attention control is critical to our daily learning behaviors, allowing us to filter the vast amount of information we are constantly faced with while remaining aware of possible changes that may occur in the environment (Focker et al., 2018). Key attention control mechanisms include focusing on a specific place, time, or object of interest, filtering out noise or distractions, and allocating attention resources in a task-optimal manner. Attention control allows flexibility to adapt as task goals or environmental demands change, and thus it is the basis for good adaptive behavior.

The relationship between video game training and attention is currently controversial, with some early studies suggesting that video games are negatively correlated with attention. Mathews et al. (2005) used functional magnetic resonance imaging (FMRI) in a controlled trial of 71 children aged 13 to 17 years to measure activation in various regions of the brain during a Stroop counting task in subjects exposed vs. not exposed to violent video game content (whether violent video games or television-mediated content over a week or a year). It was found that there were differences, between the two, those who were not exposed to violent games had higher levels of activation in prefrontal areas and those exposed to violent games had lower levels of activation in prefrontal areas, reduced cognitive control, and are more vulnerable to violent content. The study illustrates that violent games may have an effect on brain function and may be a major factor in impacting attention. Gentile and Swing et al. (2010) evaluated a sample of 1,323 children aged 6–8 years and 210 adults over a 13-month follow-up of parent-reported television and video game exposure and teacher- or child-self-reported attention problems and revealed a significant negative effect of either television or video games on attention in the different age groups.

However, there is evidence in the literature that video game training enhances various aspects of attention, including selective attention to space and time as well as attention to objects (Table 1). West et al. (2008) used the dynamic display of a monitor to present subjects with many moving “swimmer” stimuli—15 or 30 moving circles with swinging arms—in a wide field of view (circular field of 30° radius) at two levels of perceptual load. Subjects monitored this display over a period of 1.5 to 3.5 s to find the onset of the target stimulus, where one swimmer stopped moving and increased his arm swing. The results indicate that video game players outperform non-game players in spatially selected attention. In terms of temporal selective attention, studies have shown that action game training reduces reverse masking, a negative effect of attention, and as a result, action gamers are more realistic than non-gamers to perceive the timing of visual events (Mishra et al., 2011). A third aspect of attention, attention to objects, has also been shown to become better after playing video games (Green and Bavelier, 2003). That is, when performing a multi-target tracking task, a gamer can successfully track more independently moving objects and track the same number of objects at a faster rate than a non-gamer (Boot et al., 2008). Using the effect of video games on attention, Gambacorta et al. (2018) piloted a customized action video game to treat amblyopic children, with participants being assessed on visual acuity (VA), stereopsis, and reading speed after 10 and 20 h of play. It was found that video game training could influence human attention to objects and that the game method could be used as an effective adjunctive treatment for children with amblyopia, achieving an effect similar to the gold standard treatment in a relatively short period of time.

**Table 1:** Research on the relationship between video game training and attention.

**Researcher**	**Publication time**	**Experimental sample**	**Research methods**	**Experimental environment**
Mathews et al. (2005)	2005	71 children	Functional magnetic resonance imaging (FMRI) was used to measure activation in various regions of the brain during the performance of a Stroop counting task in subjects exposed vs. not exposed to violent video games (violent video games or television-mediated content with or without exposure over a week or year)	No specific description
Swing et al. (2010)	2010	1,323 children and 210 youths	A sample of 1,323 middle childhood participants were assessed during a 13-month period by parent- and child-reported television and video game exposure as well as teacher-reported attention problems. Another sample of 210 late adolescent/early adult participants provided self-reports of television exposure, video game exposure, and attention problems	No specific description
West et al. (2008)	2008	24 men with normal or corrected-to-normal vision	Using the dynamic display of the monitor, subjects were presented with a number of moving “swimmer” stimuli—15 or 30 moving circles with oscillating wire arms—in a wide field of view (circular field of 30° radius) at two levels of perceptual load. Subjects monitored this display for 1.5–3.5 s to find the onset of the target stimulus, where one swimmer stopped moving and increased its arm swing	No specific description
Mishra et al. (2011)	2011	41 youths with normal vision	Steady-state visual evoked potentials (SSVEPs)were recorded from action videogame players (VGPs) and from non-videogame players (NVGPs) during an attention-demanding task. Participants were presented with a muli-stimulus display consisting of rapid sequences of alphanumeric stimuli presented at rates of 8.6/12 Hz in the left/right peripheral visual fields, along with a central square at fixation flashing at 5.5 Hz and a letter sequence flashing at 15 Hz at an upper central location	No specific description
Boot et al. (2008)	2008	11 professional video game players and 10 non-video game players	Eleven professional video game players and 10 non-video game players were each trained in the game for 20 h, and cognitive tests were administered after the training	No specific description
Gambacorta et al. (2018)	2018	21 children with single-sided amblyopia	Participants were assessed for visual acuity (VA), stereopsis, and reading speed after 10 and 20 h of play. Additional exploratory analyses examined improvement 6–10 weeks after completion of training (follow-up)	No specific description

### 2.2. Perceptual ability

Perception is the set of processes by which consciousness perceives, senses, pays attention to, and discerns internal and external information (Lu and Ma, 2020); better perceptual skills can help people learn new skills, with one study (Chopin et al., 2019) showing that game players are about 3/4 of a standard deviation higher than non-players in all perceptual skills. The amount, type, and spatial structure of video games have an impact on the development of human perception, and relevant studies have shown that playing action video games can improve visual perceptual abilities, among others (Boot et al., 2008; Green and Bavelier, 2019), and play a reinforcing role in training visuospatial skills (Dye et al., 2009), and that action-based video game training can improve spatial cognition. The number of games and game types are major factors affecting perceptual development, and different types of video games are often accompanied by different spatial structures of the screen. Polinsky et al. examined the relationship between touch-screen video games and children's visual-spatial studying 55 children aged 3–4 years. They found that touch-screen video games were positively associated with children's visuospatial abilities (Polinsky et al., 2021). In addition, playing video games can improve skills related to game operation, especially the gamer's hand-eye coordination. With the development of electronic technology, a variety of sensors are used in electronic products and games, and children can promote their own multiple perceptual ability development when using these technologies.

Bejjanki et al. (2014) believe that the field of perceptual learning has identified changes in perceptual templates due to video game training as a powerful mechanism that leads to more regular learning and confirmed the causal role of action video games in inducing such improvements with a 50-h training study. Berard et al. (2015) found that players who played games regularly also showed a general improvement in perceptual learning, and that playing video games not only enhanced the magnitude and speed of perceptual learning but also led to faster and more stable perceptual learning. The improvements may stem from the enhanced visual perception, auditory perception, and perceptual information processing abilities of gamers compared to non-video gamers (Chopin et al., 2019).

### 2.3. Cognitive ability

Cognitive ability refers to the ability of the human brain to process, store, and extract information, that is, the ability of people to grasp the composition of things, the relationship between performance and other things, the driving force of development, and the direction of development and the basic laws (Meng et al., 2021). It is the most important psychological condition for people to successfully complete their activities. Bediou et al. (2018) showed that individuals trained in action video games improved not only on the game itself, but also on a wide range of tasks designed to develop a range of basic cognitive skills, including top-down attention, multitasking, and perception. For example, some studies have shown that playing video games improves the ability to analyze information and avoid distractions from irrelevant information during processing tasks, and this increased attention control will in turn lead to an increased ability to learn new tasks. Zhang et al. (2021) showed that individuals trained in action video games displayed faster learning in two cognitive domains (perception and working memory) tested experimentally compared to those trained in non-action games. The causal effect of action video games on learning ability was further confirmed in a pre-registered follow-up study that highlighted the increased speed of learning on new tasks as a mechanism by which action video game interventions can broadly improve task performance in the cognitive domain.

Thus, the learning transfer effect of video games is based on their comprehensive enhancement of human cognitive ability. In this regard, Cardoso-Leite et al. (2020) proposed five key game features that would guarantee the cognitive enhancement effect of video games on people (Bavelier and Green, 2019): the need to plan or make decisions under time pressure (MYBOOSTING.GG, 2022); the need to distract or maintain attention in most of the environment of a person (Aziz et al., 2021); a strong need for high precision or focused attention; and Pujol and Fenoll (2016) The need to switch between the distracted state in front and the concentrated state in the back (as required by precise aiming in shooting games, for example) according to the changing game situation. The need for prediction (activities need to be structured enough so that people can learn through iterative experimentation); (5) The need for variability (activities need to be diverse enough to avoid the automation of trained brain functions).

## 3. Mechanism and evaluation method to assess the influence of VDT workspace light environment on the rhythm system

### 3.1. Characteristics of VDT workspace light environment

Compared with the ordinary workspace light environment, the VDT workspace light environment has many typical characteristics. First, there are standard and professional training spaces for general competition events, while most of the training spaces for video games are located in independent houses or office buildings, and some of them use ordinary classrooms, without uniform standards. Most of the training spaces use the original general lighting. To avoid the reflection of the VDT screen, the windows are often shielded, so that the light environment is less interfered with by natural light, and a huge difference in brightness is formed between the VDT screen and the background (Lu and Yan, 2022).

In the VDT workspace light environment, players need to stare at the self-luminous display screen for a long time, which is the most significant feature (Gu and Han, 2005). The high intensity and focused engagement of video game training requires a high degree of hand-eye-brain coordination (Li and Wang, 2009) and visual-auditory dual-channel cooperation (Wei et al., 2014). It is difficult to separate a single factor for independent research in isolation and static mode. The complexity of the video games workspace light environment also lies in the fact that ordinary VDT operations such as typing and drawing deal with relatively slow and static information, while video game training needs to deal with fast-changing dynamic information. The mechanism is more complex, and the operation content has a greater impact on visual fatigue. In the process of routine VDT operation, the fixation rate of players on the screen has reached 60~80% (Zhang and Wang, 2010), and the player's attention rate is even higher. In addition, as a rhythm timing factor, light affects hormone secretion, circadian rhythm, sleep schedule, and other aspects of the human body (Lowden and Kecklund, 2021). Inadequate daytime light leads to a decrease in the secretion concentration of melatonin at night (Mishima et al., 2001). Inappropriate nighttime light stimulation can disrupt the normal secretion of melatonin, cortisol, and other hormones, causing rhythm disorders and sleep disorders (Boyce, 2010). However, playing for a long duration is one of the characteristics of most players (Aziz et al., 2021). Long VDT light duration and night light exposure caused by game addiction expose game players to the risk of health impacts that cannot be ignored.

The physical characteristics of the VDT workspace light environment such as color temperature, intensity, and wavelength of light may have an impact on human alertness, mood, endocrine function, and circadian rhythm (Boyce, 2010). Visual Display Terminal as a special light source is a self-luminous body in space, different from the general artificial lighting equipment, which has variable color temperatures, adjustable brightness, and dynamic unvarying display content. With the increasing scale of VDTs used for video game training, the proliferation of various VDT devices per unit space, and the change of VDT screen materials and luminescent technology (Long, 2015), VDT display lighting sources are becoming more and more important to the composition of the light environment. Display lighting sources and the physical characteristics of the light of the ambient lighting source may have a superimposing effect, such as the overall color temperature of the space may change as well as light intensity, and higher eye level illumination, resulting in the physical characteristics of the lighting parameters of the entire workspace, forming a more complex light environment for VDT workspace.

Moreover, due to the progress of display lighting and environmental lighting technology in recent years, the adjustability of lighting is gradually increasing. Compared with traditional workspaces, the light environment of the VDT workspace may have more complex adjustment mechanisms. For example, in the process of video game training, players can independently adjust the brightness of the screen and lights according to their personal preferences and habits. In addition, VDT spectral composition often contains a large amount of short-wave blue light, and its impact on the optical environment of the entire space cannot be ignored. In the case of excessive exposure, optical radiation may have potential hazards. The blue light protective glasses, anti-blue light film, low blue light display technology, and other technologies introduced to reduce the hazards make the VDT workspace light environment locally controllable and manually intervenable. Considering the possible cumulative damage caused by VDT blue light, players usually use some relevant protective equipment to reduce the light damage caused by VDT to the human body. These protective mechanisms weaken the VDT blue light as well as the ambient light entering the eyes, thus changing the player's eye position illumination, leading to changes in the impact of the light environment on the rhythm of the human body (Fu and Yan, 2022).

In short, the VDT workspace light environment is dominated by artificial lighting, supplemented by VDT display lighting, and the two interact. Given the many effects of the light environment in the VDT workplace on the work efficiency and physical and mental health of the workers (Ranasinghe et al., 2016), research on the effects of video game training “learning to learn” should pay attention to the role of the light environment.

### 3.2. Mechanism of the effect of light environment on the rhythm system in VDT workspace

**Figure 1:**
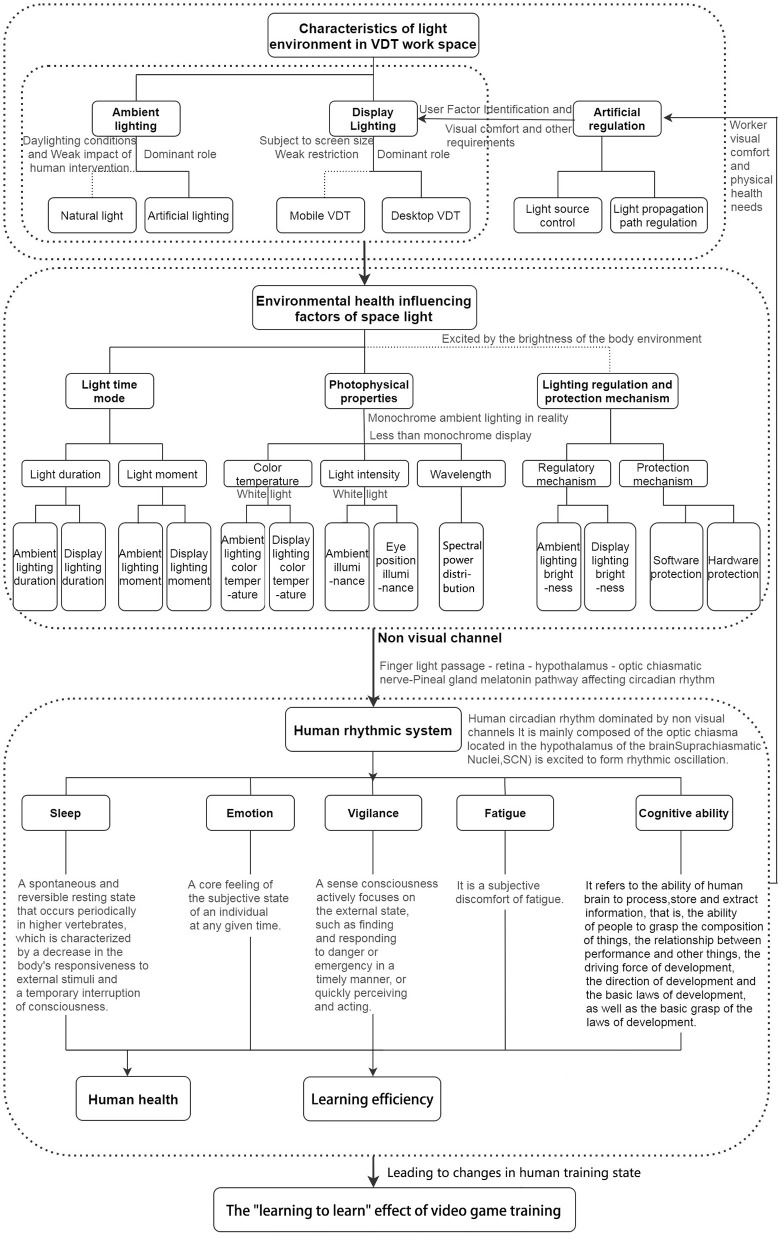
The relationship between the mechanism of working space light environment on the “learning to learn” effect of video game training.

Light passes through non-visual channels to affect human circadian rhythm, alertness, cognitive behavior, mood, etc. (Hou et al., 2021). Photobiological effects found that the non-visual effects caused by light are related to the absorption characteristics of the human body to the light of a specific wavelength, such as the duration of light, the time point of light, and the physical characteristics of light, which affect the human body's circadian rhythm system (Cai et al., 2017), thereby affecting human health and work and learning efficiency (Figure 1).

As an important timing factor, light plays a significant role in regulating and maintaining the synchronization between the internal biological rhythm of organisms and the external light and dark environment (Li et al., 2022). The study on patients with seasonal affective disorder (SAD) found that the duration of daytime light can affect their emotions. A shorter light duration in autumn and winter is the main cause of winter depression in persons with SAD (Kegel et al., 2009). Epidemiological survey results also found that the prevalence of SAD is significantly higher in high-latitude areas with relatively little year-round sunlight compared with low-latitude areas. The change in light time also affects human health, such as receiving too much light at night. A recent study based on satellite data shows that Falchi et al. (2016) more than 80% of the world's population and more than 99% of Americans and Europeans live under light pollution at night. It has been suggested that long-term exposure to night light will increase the risk of individuals suffering from emotional disorders. A recent study on a large sample (265,278 South Koreans) showed that subjects exposed to high light outdoor lights at night were more likely to suffer from depression, and depressive symptoms and suicidal tendencies were linearly related to the outdoor light intensity at night (Min and Min, 2018). The inducing effect of nighttime light on depressive symptoms shows a dose-dependent effect, that is, the longer the exposure time to nighttime light is, the more serious the depressive symptoms are An et al. (2020). Therefore, long-term exposure to night light increases the risk of depression, and the higher the night light intensity and the longer the exposure time, the more serious the depressive symptoms. The research of He and Yan (2022) shows that for assembly line workers with insufficient light, early light before work every day may have a delayed impact on participants' alertness and sleep quality. Therefore, the light time pattern has an important relationship with the healthy rhythm of human beings.

In addition to the time mode of illumination, physical characteristics such as light intensity, color temperature, or wavelength can also affect individual health (Li et al., 2022). High light intensity is significantly and positively correlated with the positive emotions of healthy people. Bright light can regulate the neural activity poles of the limbic system when processing emotional stimuli (Leach et al., 2013; Deats et al., 2014; Harb et al., 2015). Color temperature is also an important physical attribute of light. The non-visual effect of color temperature on emotion mainly depends on the blue light component in the spectrum. The higher the color temperature, the shorter the wavelength of the blue light content in the spectrum is, and vice versa. Although there are relatively few studies on the effect of color temperature on emotional nonvisual effects alone at present, and the differences in exposure duration, research objects (daytime human/nighttime mouse), measurement indicators, etc. of different studies lead to nonconformity, it can be shown that color temperature can affect human emotion and fatigue. A recent study (He and Yan, 2022) found that for assembly line workers, higher illuminance was associated with improved subjective work alertness and higher sleep efficiency, while lower color temperature slightly improved alertness. At the same time, the dynamic changes of the light environment in the VDT operation and the blue light protection measures also change the impact of the light environment on the human rhythmic system.

To sum up, due to the particularity of the video game operations, VDT equipment plays an obvious role in the formation of the VDT workspace light environment. Compared with the traditional operation forms, the effect of VDT display lighting on the non-visual effects of video game operators cannot be ignored.

### 3.3. Evaluation method of the effect of light environment on the rhythm system in the VDT workspace

The current evaluation model of non-visual effects of light environment is mainly based on the spectral response curve of non-visual effects to evaluate the non-visual effects in buildings (Zhang et al., 2020). The methods can be broadly divided into two categories: one to evaluate the static light environment, and the other to use computer simulation methods to evaluate the dynamic natural light environment in the building space, such as weighted daylight autonomy (DA) value (Pechacek et al., 2008), Capped graph (Andersen et al., 2012), and the circadian effective area (CEA). The influencing factors that have been considered in the evaluation model of non-visual effects of the light environment in general places mainly include spectral distribution, light intensity, light duration, light moment, and personal illuminated history.

Various non-visual effect evaluation methods also have different considerations on these influencing factors, and their advantages and disadvantages are also different. However, there are some correlations between these evaluation methods, which promote the development and improvement of daylight evaluation methods based on non-visual effects. Although the current research on the non-visual effects of light is not enough to give some exact values to meet people's demand for non-visual effects in the design of building lighting, these evaluation methods provide us with ideas to measure the non-visual effects of lighting and building a framework that can be developed sustainably (Table 2) (Jiang and Wu, 2018).

**Table 2:** Summary of evaluation methods examining the non-visual effects of light.

**Evaluation method**	**Main feature**	**Year**	**Influencing factors of non-visual effects**					**Evaluation object**
			**Spectral distribution**	**Light intensity**	**Light duration**	**Light moment**	**Illuminated history**	
Rhythm influence factor C/*P*-value (Maki and Adams, 2004)	Evaluate the non-visual effects produced by static light environment through actual measurement and calculation	2004	Yes	Yes	No	No	No	Light sources
Biorhythm stimulating factor CS value (Rea et al., 2005)		2005	Yes	Yes	No	No	No	Light sources and architectural space
Light dose method (Ju et al., 2012)		2012	Yes	Yes	Yes	No	No	Light sources
Weighted DA value (Pechacek et al., 2008)	Computer simulation is used to evaluate the non-visual effects produced by the dynamic natural light environment in the building	2008	Yes	Yes	No	No	No	Architectural space
Capped graph (Andersen et al., 2012)		2012	Yes	Yes	Yes	Yes	No	Architectural space
Percentage of effective circadian rhythm area CEA (Konis, 2017)		2017	Yes	Yes	Yes	Yes	Yes	Architectural space

However, there is no targeted evaluation model which can appraise the non-visual effects of the VDT workspace light environment. Based on the existing evaluation model and the known impact mechanisms of VDT on human health, the elements and objects in the existing evaluation model can be improved to form a more targeted evaluation approach. For example, based on the “effective circadian rhythm area percentage CEA evaluation method” (Konis, 2017) proposed in 2017 for improvement, more attention can be paid to displaying lighting effects in the evaluation process, and spectral measurements can be conducted separately for the common modes of VDT operations in building spaces. When VDT is normally on without ambient lighting, when VDT is off with ambient lighting, and when both are simultaneously on, and the light hours and light moments of VDT can also be considered separately, then a model for evaluating the non-visual effects of the light environment that is relatively suitable for VDT operation spaces can be deduced.

## 4. Influence of VDT display lighting on the function of the special impact of the workspace light environment

In terms of the effect of light timing patterns on human rhythm health, studies in the fields of architectural design and gaming, where VDTs are deeply applied, have found that the duration of VDT display lighting and light moments affect human health rhythms (Nakazawa et al., 2002; Cheng et al., 2019; Zhang and Yan, 2019). In addition, in terms of the photophysical properties of display lighting, studies in the area of color temperature (Yang et al., 2017b; Zhou and Zhou, 2019) have demonstrated that the display color temperature of the VDTs can result in different blue light hazard factors and rhythm effect intensities. In the real situation, due to the use and power limitations of conventional desktop and mobile VDTs, luminous intensity is low as is the chance of displaying monochromatic images, so the influence of the luminous intensity of VDT and the spectral distribution of monochromatic display is only briefly discussed in this section. In addition, as a special light source in space, VDTs can also cause changes in the overall working space light environment due to their special regulation and protection mechanisms, which can affect human health rhythm. Therefore, for video game training, which is highly dependent on VDT equipment, the impact of its light environment on the human body is more special than that of general work, and it is very likely to be related to the impact of VDT display lighting.

### 4.1. VDT light time mode function

#### 4.1.1. Light duration

Human light health is influenced by the length of time it is exposed to the light (Li et al., 2022), and the effects of VDT blue light; non-visual effects also change according to the duration of exposure. At the same time, the VDT mode of operation has a variety of types. Different operation types have different continuous operation times which may cause the human body to be affected by the VDT display lighting duration differently, which in turn leads to the similarities and differences in the impact of the light environment on human health (Table 3).

**Table 3:** Effect of VDT illumination duration on human health.

**Investigator**	**VDT task type**	**Daily VDT light duration**	**Aspects of VDT on human health effects covered by the study**
Nakazawa et al. (2002)	Ordinary copywriting office	5 h	Spirit, sleep
Cheng et al. (2019)	Internet IT operation	11 h	Job burnout, depression
Zhang and Yan (2019)	Design graphics	>8 h	Far and near vision, color vision, karolinska sleepiness scale (KSS) distress, brief profile of mood state (BPOMS) mood, ocular surface disease index (OSDI) dry eye

One study (Nakazawa et al., 2002) addressing the effects of VDT display lighting duration on human health was based on a survey of more than 25,000 office workers, and a factor analysis was conducted to extract three factors: psychological, physical, and sleep-related symptoms. The adjusted mean of each factor score was compared to the duration of each VDT use by a general linear model. It was found that physical symptom scores became higher as the daily duration of VDT use increased. Psychological and sleep-related symptom scores were significantly higher for workers who used VDT for more than 5 h/day than in the groups that used VDT for more than 1, 1–3, and 3–5 h/day. The study also found that the duration of daily VDT use was linearly related to physical symptom scores and non-linearly related to mental and sleep-related symptom scores, with a threshold effect of 5 h/day.

A subsequent study (Maki and Adams, 2004) explored longer-duration VDT work and its association with physical and mental symptoms of difficulties. A long-term study of 944 Internet employees by Maki and Adams (2004) found that single sustained use of VDT for >4 h per day and cumulative hours >11 h per day had adverse effects on the physical and mental status of office workers. The study showed that extended VDT hours significantly increased the risk of back pain, wrist pain, hip pain, dry eye, eye soreness, burnout, and high occupational stress, and showed that people who used VDT for more than 4 h a day were at higher risk for depression. Among the above effects, back pain, wrist pain, and hip pain may be mainly due to non-light environment effects such as the improper posture of VDT operators (e.g., leaning forward) or improper workstation setup; however, job burnout and depression are most likely induced by the human body due to excessive display illumination light.

Some industries that are highly dependent on VDT, such as the construction industry, involving conceptualization, modeling, drafting, rendering, and layout of VDT operations, require frequent switching between multiple types of software and scenes and require a large number of complex eye movements (Su and Liu, 2016; Yao and Miao, 2016). Frequent adjustment and vergence movements (McLean et al., 2001) of the operator's visual system are also needed, making the use of VDT equipment more intensive than that of a single job type. In addition, it has been shown Yuan and He (2012) that excessive light exposure can cause retinal photoreceptor and pigment epithelial (RPE) cell damage and macular degeneration. Repeated light exposure can also cause cumulative retinal damage, and long-term low-intensity intermittent visible light exposure accelerates the aging process of the retinal macula (Bi, 2014). The blue light component is the most damaging to the retina and crystal and is an important blinding factor for macular degeneration.

One of the most important blindness-causing factors is macular degeneration; visual fatigue may be associated with the current early age of onset and increased prevalence of certain chronic eye and retinal diseases (Zhao and Huang, 2016). Another study (Zhang and Yan, 2021) divided 16 subjects into two body types based on the Traditional Chinese Medicine Body Theory and analyzed the data of various states and performances before and after performing a 1-h construction-type VDT operation using SPSS software. The study showed that for both types of subjects, the performance of distance and near visual acuity, color vision, KSS sleepiness, BPOMS mood, and OSDI dry eye decreased after the VDT operation, but there were differences in the extent of the effects according to body type. Thus, high-load VDT operations, such as design and drafting operations, have more severe effects on body rhythms due to light exposure caused by the high duration of display lighting compared with ordinary operations.

#### 4.1.2. Light moment

Before the invention of artificial lighting, the human light pattern was a light-dark cycle close to 12~12h. With the invention of electric light, the widespread use of VDT, night shifts, shift work, and travel across time zones became common, and the moment of light received by humans changed. This is not only because the human “sunrise and work, sunset and rest” work habits have different time lighting requirements, but also because the human body rhythm, body temperature, and hormone levels have a cycle of changes in the day and night cycle (IEC, 2014). In this regard, the German technical specification DIN SPEC 67600 also recommends the use of a dynamic light scheme close to the natural light change patterns in the office to meet the light requirements of people at different times (Table 4).

**Table 4:** Light moment characteristics of various types of operations.

**Task type**	**Type of light sources**	**Illumination time**	**Feature**
Traditional surface task	Natural light and artificial lighting	Daytime routine office hours	It is greatly affected by natural light and has little effect on normal human rhythm (Lin et al., 2018).
Routine VDT office task	Artificial lighting and display lighting	Daytime routine office hours	Affected by the superposition of multiple light sources, human rhythm is affected to a certain extent (Gao and Yan, 2020).
High-intensity VDT operations such as video game training	Artificial lighting and display lighting	Any time of the day	A large amount of artificial light at night is easy to affect human emotions and sleep quality (Zhang and Yan, 2021).

The time point of light exposure is significantly and negatively associated with depressed mood; the earlier the morning exposure to light (McLean et al., 2001; Su and Liu, 2016), the lower the depressed mood score. Video game training is often conducted in darker ambient lighting, players are often exposed to light at random points in time, and often work intensely at night, and such extreme light patterns may increase the incidence of depressed mood. At present, due to the short period of time that research on the light environment of video game training has been conducted and the lack of relevant research data, it is still necessary to be alert to the effects of its light moments on the rhythm system.

### 4.2. The function of VDT display illumination light's physical property

#### 4.2.1. Display color temperature

The human body's intrinsically photosensitive retinal ganglion cells (ipRGCs), which contain a photopigment called melanopsin, play an important role in the production of blue light (Shen et al., 2021), and the blue light content often has an important connection with the color temperature of the light source. LED is currently the most common backlight source for VDT screens. Most of the current LED backlight screens can be adjusted for color temperature, and the difference in spectral distribution at different color temperatures is considerable (Feng et al., 2015; Zhu et al., 2017). In terms of the impact of the physical properties of VDT light, it has been found that the percentage of blue light from LED backlight screens increases with color temperature (Algvere et al., 2006) and that the blue light hazard factor also increases rapidly with color temperature. Ma et al. (2017) conducted a screen spectrum test on three mainstream models of LED computers, and the spectrum was generally in the visible range of 400-780 nm. The blue light peak of the two screens was at 450 nm, and the blue light peak of the third was at 436 nm and 487 nm, respectively. One of the peaks was exactly at the blue light hazard weighting function B(λ), and its wavelength is close to the strongest wavelength of blue light hazard indicating that the luminous spectrum of the computer screen was not safe.

Studies have already started to explore the effects of different VDT color temperatures on the photobiological effects in humans. This type of research is mainly based on the expression of dark-to-light ratio Scotopic/Photopic(S/P) to quantitative assessment of blue light hazards by the Blue Light Hazard Factor (K_B_) in IEC/TR62778-2014 (IEC, 2014) and quantification of the intensity of rhythm effects by the Rhythm Factor (K_C_).

Using the above method Yang et al. (2017a) compared blue light hazards and non-visual biological effects of LED backlight screens with different color temperatures. The study combined the transmittance capacity of human eyes of different ages and used MATLAB software to investigate the changes of illuminance factor, blue light hazard factor, and rhythm factor with the color temperature of the backlight screen. The results showed that the percentage of blue light of the LED backlight screen increased with the increase in color temperature (1,200–6,500 K), and the illuminance factor decreased with the increase in color temperature and user age. It was more influenced by age. The blue light hazard factor increased rapidly with the increase of color temperature and decreased with the increase of users' age. The rhythm factor increased rapidly with the increase of color temperature and decreased with the increase of the user's age.

However, the above methods require parameters such as actual measured spectral distribution data, in addition to the four response function expressions of the human eye, and fitting the response function calculation, which is a more complicated process. Therefore, subsequent studies have tended to simplify the computational model according to the characteristics of the study object and to analyze the study with approximate results. For example, in the study of the light health effects of VDT, combined with the visible blue light hazard weighting factor function B (λ), most of the VDT screen blue wavelength (95.6%) is in the 400-500 nm band, so the blue light hazard weighting factor can be approximated as 1 in the range of 400-500 nm, and the other bands ignored as 0. Therefore, a simplified model was established with the percentage of blue light from 400 to 500 nm, and the model was used to find the *R*_*B*_ (Approximation of *K*_*B*_ in the limited wavelength band) value instead of the visible wavelength (380–780 nm) blue light hazard factor, as an approximate characterization of the strength of the blue light hazard. Similarly, since the rhythm function C (λ) is mainly located in the 446-477 nm band, the 446-477 nm blue light was approximated as 1, and the R_C_ (Approximation of *K*_*C*_ in the limited wavelength band) value derived from the simplified model was used to approximate the strength of the rhythm effect.

Using a simplified computational model as the basic experimental principle, Yang et al. (2017b) conducted a comparative study on the blue light hazard and rhythm effects of different displays. The study used the standard light source D50 (5,000 K) defined by ISO 3664-2000 as the white field color temperature for the test, and the four commonly used displays. They consisted of a cold cathode fluorescent tube (CCFL)-backlit liquid crystal display (LCD), light emitting diode (LED)-backlit LCD, organic light emitting diode (OLED), and cathode ray tube (CRT) and were measured at this ambient color temperature in displaying the spectral distribution of the color temperature at 1.200, 1,900, 2,300, 2,700, 3,400, 4,100, 5,000, and 6,500 K. The S/P, R_B_, and R_C_ values of the four displays at different color temperatures were calculated from the measured results, and the S/P, R_B_, and R_C_ values of the four displays increased with the increase in color temperature.

Given that the working color temperature of the display in the real working scenario is usually above 5,000 K, the experiment also studied the effect of the spectrum under 6,500 K working conditions. The results showed that when the screen color temperature was 6,500 K, the RB value of CRT and CCFL-backlit LCD were both around 40% higher than the 32% of LED-backlit LCD and OLED. That is, the blue light hazard of LED-backlit LCD and OLED screens was less than that of CRT and CCFL-backlit LCD. The RC value of both CRT and LED-backlit LCD was about 18%, which was 10% higher than that of OLED; that is, the comprehensive performance of the OLED screen is the best under these conditions.

The study also calculated the blue light hazard and rhythm factors of the four displays at different color temperatures using the complex conventional method and compared and analyzed the results with the simplified calculation to verify its validity. The validation experiments showed that the R_B_ value and the blue light hazard factor K_B_ differed by only one proportional constant, and that the R_B_ value could be used to study the blue light hazard instead of the blue light hazard factor K_B_ for the four monitors tested in the color temperature range of 1,200–6,500 K. However, no obvious correlation was found between the R_C_ value and the rhythm factor K_C_, so it could not be used to approximate the change of the rhythm effect with color temperature for the four monitors.

In terms of the impact of the display color temperature of removable VDT devices in VDT operating spaces, relying on simplified computational models, the blue light hazards and rhythm effects of three common materials of cell phone screens have also been studied (Zhou and Zhou, 2019) (LED-backlit LCD, OLED, SUPER AMOLED) in the commonly used operating color temperature range (above 7,000 K). Four experimental selections of color temperature were measured (7,425, 8,091, 8,309, and 9,201 K). The results showed that both the rhythm factor and the blue light hazard factor of the tested cell phone increased with increasing color temperature, and both showed a good linear relationship. For different types of screens with color temperatures ranging from 7,425 to 9,201 K, the rhythm factors are, in descending order, OLED > SUPER AMOLED > LED-backlit LCD, and the blue light hazard factors are, also in descending order, LED-backlit LCD > SUPER AMOLED > OLED. The blue light hazard factor ranking based on the simplified model is of a reference value.

Therefore, the color temperature of VDT display lighting coupled with the ambient lighting color temperature has a certain impact on the light health elements of the VDT operating space light environment, which in turn affects human health (Table 5).

**Table 5:** Display the effect of color temperature on human health.

**Investigator**	**Experimental research methods**	**Research object**	**Primary variable**	**Conclusion**
Yang et al. (2017b)	The spectral distribution of LED backlight screens with different color temperatures was measured, and multiple function expressions were fitted with high-quality fitting according to the relevant original data.	Desktop VDT	User age:	The proportion of blue light in LED backlight screen increases with the increase of color temperature, and the illuminance factor decreases with the increase of color temperature and user age and is more affected by age. The blue light hazard factor increases rapidly with the increase of color temperature and decreases with the increase of user age. Rhythm factor increases rapidly with the increase of color temperature and decreases with the increase of user age.
			Youths: aged over 20-year-old	
			Old: aged between 40 and 50-year-old	
			Color temperature:	
			1,200, 1,900, 2,300, 2,700, 3,400, 4,100, 5,000, and 6,500 K	
Yang et al. (2017a)	Based on the expression of S/P and the spectral characteristics of VDT screen, the calculation model of blue light hazard weighting factor function B (λ) and rhythm function C (λ) is simplified	Mobile VDT	Screen material:	When the color temperature is 6,500 K, considering the harm of blue light and the rhythm effect, the advantages of the four displays, the ranking from best to worst is LED backlight, LCD, OLED, CCFL backlight, LCD, VRT.
			Cold cathode fluorescent tube (CCFL) backlight, Liquid crystal display (LCD)LED backlight, LCD, *Organic Light-Emitting Diode* (OLED), *Cathode Ray Tube* (CRT)	
			Color temperature:	
			1,200, 1,900, 2,300, 2,700, 3,400, 4,100, 5,000, and 6,500 K	
Zhou and Zhou (2019)		Mobile VDT	Screen material:	The blue light hazard factors of all kinds of screens increase with the increase of color temperature, and the overall performance of the OLED screen is the best.
			LED backlight, LCD, OLED, SUPER AMOLED.	
			Color temperature:	
			7,425, 8,091, 8,309, and 9,201 K	

#### 4.2.2. Light intensity and spectral distribution

Light intensity is an important factor influencing non-visual effects, and early experimental data from Brainard, Thapan, and Rea showed a significant increase in the inhibitory effect on melatonin secretion with increasing light intensity (Brainard et al., 2001; Thapan et al., 2001; Rea et al., 2002). In recent years, domestic and international studies have focused on the effects of lighting intensity on sleep, productivity, and alertness. For example, Huiberts found that subjects performed significantly better on test tasks at 1,700 lux compared to 165 lux (Huiberts et al., 2016). Relevant studies have also confirmed that under the high-illumination stimulation of 750~1,000 lx, students' fatigue was significantly reduced and learning efficiency improved (Yan et al., 2012; Guan and Yan, 2019). From the above studies, it can be determined that high illumination is needed to stimulate the human rhythm effect. The limited luminous intensity of common indoor desktop VDT or mobile VDT devices may have some effect on rhythm health, but the extent of the effect remains to be explored. A recent study of medical instrument displays (LCDs) (Yoshimura et al., 2020) found that the amount of blue light was proportional to the brightness of the display and that reducing the brightness of the display did not significantly reduce the visibility of the display, but could reduce blue light at peak wavelengths by about 56%.

Rao et al. (2015) let VDTs display pure blue and white pictures respectively, and further studied them in combination with the transmission spectrum of the eye lens at different ages. In this study, the spectral distribution P(λ) of four different models of LED-backlit displays was measured, and the retinal illumination factor, rhythm factor, and blue light hazard energy efficiency factor were calculated for human eyes of different ages. They found that the retinal illuminance factor, rhythm factor, and blue light hazard energy efficiency factor of common LED-backlit displays, changed with age, and for the same display, whether it was displaying blue or white pictures, these parameters decreased rapidly with increasing age. For older adults and young people, the differences in the illumination factors of different monitors were small for the same age group of human eyes. However, for young people, the difference in rhythm factor and blue light hazard energy efficiency factor values was greater for different monitors, but the difference was smaller for older adults.

The above study confirms that the intensity and spectral distribution of VDT display lighting have some effect on the human body, although the effect is relatively mild compared to other factors. However, this effect is most likely to superimpose with the photobiological effect of other lighting sources in the VDT workspace, and then have an effect on the “learning to learn” effect of video game training.

### 4.3. VDT display lighting regulation and protection mechanism functions

#### 4.3.1. Regulation mechanism

The more balanced the brightness distribution in the field of view, the lower the degree of visual fatigue of office workers and the stronger the subjective comfort. If the illumination of the surrounding space changes abruptly, it is easy to cause visual discomfort for office workers. In the past, due to economic and lighting product limitations, the traditional conventional office space light environment was mainly ambient lighting and natural light with limited possibility of dynamic adjustment. In recent years, LED lighting products with adjustable brightness and color temperature (Jung et al., 2017) and VDTs and other electronic products with strong adjustable parameters and dynamic displays have become popular, resulting in the light environment of the VDT workspace beginning to be dominated by artificial light (Gao and Yan, 2020). Moreover, the interaction between ambient lighting and display lighting makes the spatial light environment adjustment mechanism more changeable and complex.

In recent years, for VDT and the interaction of ambient lighting on spatial light distribution, many scholars have researched the intelligent regulation of LED lighting and the creation of personalized light environments, resulting in a large number of research findings (Shi and Lin, 2020; Li et al., 2021). For instance, in terms of the VDT operating space lighting environment, subjective operator preference research by Yu and Akita (2020) experimentally demonstrated that the ambient luminance and screen luminance ratio during VDT visual operations also had a large impact on comfort, and determined the comfort range of screen luminance at different ambient luminance. Similarly, de Bakker et al. (2018) conducted a subjective evaluation study of the relationship between the luminance level and the luminance ratio between the operational task area, the adjacent surrounding area, and the background area of an open VDT office and found that the overall luminance was either too high or too low to be desirable. The comfort level of the integral luminance was highest when the task area luminance was close to the neighboring surrounding environment luminance and the task area luminance was about two times the background luminance. In addition, it was found that if users were not satisfied with both work surface illuminance and screen brightness, no form of external lighting could be rated as satisfactory. In other research, Chraibi et al. (2017) compared the relationship between wall brightness and preferences regarding VDT work surface illuminance. The background brightness level across the field of view determined the subjects' preferred choice for horizontal illumination of the work surface, and wall background brightness was inversely related to the choice of work surface illumination. Luo and Yan (2021) compared the effects of different dimming modes in an open office on various visual physiological and psychological indicators of young VDT operators and found that the indicators were significantly better in the personal preference light environment than in the conflicting (non-preference) light environment.

In a study of the multi-person collaborative dimming model for the VDT workspace, Luo (2020) pointed out that the screen brightness adjustment of VDT was generally based on the detection of ambient brightness by sensors and also by manual adjustment by people. The influencing factors were mainly ambient light, user state, and the scenarios behind the application. Despenic et al. (2017) experimentally explored a multi-person collaborative consensus control model for VDT in an open office with shared work areas and established a light environment preference profile model based on user control behavior and preference information. However, they did not propose a specific implementation plan. Chraibi et al. (2017) pointed out that using low illuminance as a starting point can reduce the illuminance preference value of the work surface and reduce the illuminance conflict between adjacent workstations.

Dynamic lighting is the basis for ensuring that the VDT workspace light environment can meet subjective preferences and achieve collaborative dimming mode in an environment with multiple people. Zhu and Duan (2022) and others studied a dynamic lighting system based on the Hybrid Particle Swarm Firefly Algorithm (HPSFA), with energy savings of at least 13% compared to traditional lighting and 2% compared to other algorithms. Zhu's study shows that the dynamic lighting model can significantly improve the comfort and satisfaction of VDT workspace personnel, and thus has some practical application value.

Therefore, the VDT workspace light environment, due to the existence of special light sources, ambient lighting, and display lighting, forms an interactive two-way adjustment mechanism, so that the overall light environment is more complex and variable. The impact of this type of complex light environment on the health of people in space is yet to be explored.

#### 4.3.2. Protection mechanisms

Recent studies have shown that a moderate amount of blue light is necessary to participate in adolescent refractive development (Zhao and Huang, 2016), but for VDT operations, blue light illumination in normal operation can cause retinal damage without extreme experimental conditions (Lappe, 2017). Current VDT display lighting protection mechanisms are mainly interventions against its harmful blue light (high-energy short-wave blue light from 420 to 460 nm). However, such methods usually also affect eye level illumination at the same time, so the photobiological effect of the VDT light environment on the human body is changed. At present, anti-blue light technology can be divided into two categories: anti-blue light software technology and anti-blue light hardware technology.

**Figure 2:**
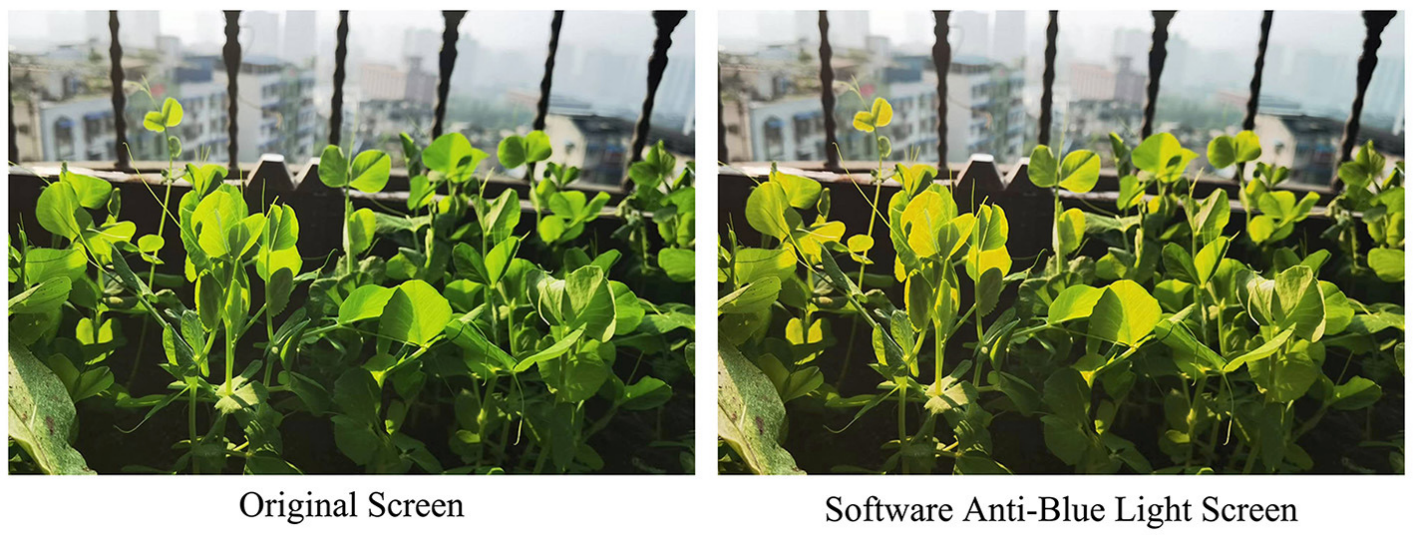
Display color cast caused by anti-blue light technology software.

Anti-blue light software technology mainly relies on the eye protection mode, the so-called night mode to achieve blue light protection. The principle is to limit the blue light content in the RGB three-color channel in VDT through a specific software algorithm and reduce the user's exposure to the blue light generated by the display device. The advantage of this technology is that it is user-friendly, and the software can be turned on or off according to the application scenario. The disadvantage is that the “one-size-fits-all” working mechanism leads to a reduction of blue light in all wavelengths, resulting in severe color bias on the screen (Figure 2), reduced brightness and contrast, and reduced clarity of text and images, which can still cause eye damage over time (Li and Wang, 2019).

Anti-blue light hardware technology mainly focuses on controlling the emission source of blue light and blocking it during propagation. In terms of VDT blue light emission source control, Li and Wang (2019) proposed two hardware measures: the first was replacing advanced LED chips and fine-tuning the LED light source to migrate the peak position of harmful blue light from <450 to 460 nm. The second measure was achieving an anti-blue light effect by applying a blue light filter (including tempered film) to the surface of display devices or wearing anti-blue light glasses to produce an anti-blue light effect.

Wang et al. (2020) examined the development directions of anti-blue light display technology, first by increasing the wavelength of the blue light chip, which is similar to the method proposed by Li and Wang (2019). However, Wang et al. further pointed out the limitations of this method in addition to its high cost; for example, it will also affect other optical parameters such as the display color gamut. Second, they used candlelight OLED technology which is a new OLED material that can obtain candle-like spectra. Because the candlelight spectrum is close to the standard light source, its spectral intensity increases with increasing wavelength. Its yellow-red light ratio is larger, and the blue light ratio is the smallest, so the blue light is less harmful. Third, Wang et al. added a blue light reflective film layer to VDT, based on the principle of thin-film optics. SiO_2_ and SiN film layers with different refractive indices were affixed alternately on the display panel, and by controlling the thickness and number of layers of the two films, the reflection of specific wavelengths could be achieved to prohibit their transmission out of the display panel. In terms of hardware technology for blocking the propagation process of VDT blue light, blue light protection using glasses is of current focus. Yang et al. (2018) compared the filtering effect of three different colors, processes, and materials of anti-blue light glasses on-screen blue light, finding that the anti-blue light glasses with yellow resin material had the best effect, filtering 90% of blue light; the remaining lenses filtered blue light between 40 and 60%.

Although the above VDT display lighting protection mechanisms can provide some control over the risk of blue light hazards, all these mechanisms have imperfections. Anti-blue light software technology changes the display brightness of the display, reducing the amount of light from the display lighting to the human eye. Anti-blue light hardware technology filters light with a lens coating layer, reducing the amount of light from display lighting and ambient lighting to the human eye. Eye-level illumination is an important indicator of light health, however, whether the reduction of eye-level illumination will have other effects also need to be explored.

## 5. Discussion

With the “learning to learn” effect of video game training being explored increasingly, video games are not only a tool for entertainment and leisure in people's lives; their attributes as a learning ability enhancement aid are being strengthened continuously. But how to optimize the “learning to learn” effect of video game training, in addition to the exploration of game content, training mode, game equipment, and other aspects, the optimization of the training environment is also a very valuable research direction.

The light environment has a huge potential impact on the efficiency of indoor operations, including not only the ability of indoor personnel to perform visual tasks but also the comfort of personnel and various physiological functions related to the rhythm system. There has been relatively little research on the specific effects of the typical VDT workspace light environment on human rhythm health. Although research on the effects of VDT display lighting has gradually begun to receive attention, existing studies have shown that the temporal patterns of VDT operations, photophysical properties of the halo adjustment mechanism, and protection mechanisms can affect the rhythm system of the operator. However, is still unclear whether the effect of this kind of light environment on the human rhythm system for the effect of video game training “learning to learn” has a facilitating effect or an interference effect. How to create a light environment conducive to the “learning to learn” effect also needs to be explored.

Therefore, it is important to accurately and comprehensively study the photobiological effects of the light environment in the VDT workspace, so as to identify the correlation between its key influences on the effect of “learning to learn” (Algvere et al., 2006).

### 5.1. Some considerations for the light environment of the VDT workspace

The non-visual effects of the light environment of the VDT workspace can have an important impact on human health, work efficiency, and emotion, and its optimization is of great significance to improve the “learning to learn” effect of video game training. In the real environment, it is often difficult to reduce the length of light exposure by limiting or reducing the length of VDT use due to productivity requirements. Consequently, the harmful effects of VDT can be reduced by selecting VDT screen materials and adjusting parameters such as screen color temperature without changing operating hours. In terms of the rhythmic interference of VDT light moments to personnel in the operating space, for night operations that are difficult to circumvent, the use of high-brightness VDT equipment in the dark should be avoided, and the use of ambient lighting aided by appropriate brightness is appropriate.

In terms of blue light protection in VDT workspace, on the one hand, office workers face a greater potential risk of retinal blue light damage during long hours of VDT operations, and the problem of visual fatigue caused by screen blue light is prominent. On the other hand, the necessary blue light can improve the degree of alertness and picture color recognition during operations, which has a positive effect on maintaining work efficiency. Thus, research on the effect of VDT blue light on human rhythm health should explore the reasonable range of blue light shielding and shielding ratio to achieve normal work conditions based on ensuring the reduction of operational visual fatigue and the potential risk of blue light hazard.

The VDT office space light environment is different from the traditional desktop environment, mainly because VDT is a self-luminous body. The screen brightness, color, contrast, and other display parameters can be adjusted according to the surrounding environment and the subjective feelings of office workers. To a certain extent, this flexibility can make up for the defects of the ambient lighting, so that office workers' natural light needs can be reduced. The introduction of natural light can reduce lighting energy consumption and positively impact the human mental state, psychological feelings, and rhythmic rest. The VDT office space light environment should focus on the use of natural light or use simulated sunlight spectrum for lighting. Also, too high a difference in brightness between the VDT screen and the background should be avoided when training at night.

Ambient lighting is an important component of the VDT operating space light environment, and its design should be adjusted and upgraded according to the characteristics of VDT operations and the individual differences of users. For example, the ability to combine VDT display lighting parameters during operation and dynamically adjust the brightness and spectrum to reduce the overall light environment to bring health benefits to the operators would be a worthy objective. Ambient lighting design can even be combined with intelligent lighting control technology to create a comfortable and healthy VDT operating environment. Although the additional hardware and computing devices in the lighting system could increase costs and energy consumption, when one considers the hidden economic value brought by better personnel health, it's cost and performance would be much better than the traditional lighting methods.

Overall, the VDT workspace lighting system needs to have certain spectral and human health indicators monitoring capabilities to address the non-visual outcomes. Consideration should be given to individual human differences to accomplish the goal of creating a light environment that can improve the “learning to learn” effect of video game training.

### 5.2. Future research direction of VDT workspace light environment

The current VDT workspace light environment related research has not been able to systematically explain the mode and degree of influence of display lighting on the light health indicators of various types used to monitor the operating space. There are often no ambient lighting conditions, there is long-term use of VDT equipment and varying operating scenarios. The existing VDT lighting hours on human health research in the experimental process did not completely strip the ambient lighting. Research focusing only on the display lighting light duration and light moment health and their impact is less; future research needs to explore this further.

In addition, although the benefits of VDT using low color temperature display have been established, due to the color realism of the display screen, it is difficult to greatly reduce the color temperature as it can only float within a limited range (±1,000 K). How to manage the color temperature to reduce the blue light hazard is worthy of further research. Even if the color temperature remains the same, the impact of different photobiological effects of different screen materials will be different. Therefore, future research on healthier VDT screen materials is also a point of great concern.

Desktop and mobile VDT devices are limited by function, power, size, and luminous intensity, and the impact of light intensity changes on the rhythm of the human body is rarely studied. However, all kinds of large screens have been introduced to the operating space now, including VDTs of large areas, high luminance, and long running time. The luminance of some large screens is no less than the lamps used for ambient lighting. Therefore, for the future evaluation of the light environment quality of office space, the influence of the light intensity of various types of VDT screens should not be ignored.

While the properties of VDT itself have many implications for the health of the light environment in the workspace, individual differences in operators, such as age, gender, light history, visual deficits, stressors, and other factors, can also contribute to the similarities and differences in the effects on the circadian rhythm system. Tkatchenko et al. (2013) described the dependence of the human eye on the long-term light environment, which, when faced with changing and poor light, may generate incorrect visual signals, causing abnormalities in the eye and visual system and leading to the development of related eye diseases such as myopia. Among the many factors influencing individual differences, the effect of age has been extensively confirmed by available studies (Yang et al., 2017a,b). Factors other than age have only been more tentatively explored, and human adaptation to artificial light should be linked to individual genes and the light environment to which the person is adapted during growth, i.e., it contains two major elements of congenital and acquired influences, but the specific effects are unclear. For example, some studies suspect that human tolerance and preference for artificial light seems to be related to the light environment where one grows up, and to gender, age, and the current condition of the individual person. Those studies have attempted to explain it using the Chinese medical theory of constitution (Zhang and Yan, 2019, 2021). However, there are still relatively few existing studies, and there are still many questions that need to be explored about the similarities and differences in the effects of the VDT workspace light environment on human health, considering individual differences and preferences. At present, there are still many unanswered questions about the “learning to learn” effect of the light environment on video game training in the VDT workspace. For example, do individuals with different basic conditions have different training effects under the same training intensity of video games due to different preferences for the light environment? Furthermore, for groups with poor game training effects, whether suitable light environment patterns can be found to improve the training effects needs further study.

## 6. Conclusion

With the continuous exploration and urgent demand for the “learning to learn” effect of electronic games, people's demands for the light environment of training space can also expand from meeting the needs of visual work to emotional regulation, sleep quality, environmental cognition, rhythm repair, and other aspects. As one of the important environmental factors affecting the “learning to learn” effect of video games, the VDT workspace light environment is developing in the direction of both group synergy and high individual adaptability, to realize the gaining effect of video game training. Although the lighting control technology and display technology, which are important components of the light environment in VDT workspace are maturing, how to use them more reasonably to achieve the optimal gain effect of the light environment in this kind of space needs more scientific and engineering verification.

## Author contributions

XC completed the theme and overall writing of this review. YY is the general director of this research project, and determines the main direction of this paper. TH is responsible for the translation of tables and the collation of references in this article. YL and YZ is responsible for the picture translation and reference collation of this article. All authors contributed to the article and approved the submitted version.
